# Consideration of T-Cell Profile in the Examination of Statin Efficacy in Inflammatory Diseases, Neurodegeneration, and Neurocognitive Performance

**DOI:** 10.1093/gerona/glae156

**Published:** 2024-07-04

**Authors:** Matthew A Hintermayer, Daniel Mendelson, Jae Hyun Byun

**Affiliations:** Montreal Neurological Institute, McGill University, Montreal, Québec, Canada; Faculty of Medicine and Health Sciences, McGill University, Montreal, Québec, Canada; Faculty of Medicine and Health Sciences, McGill University, Montreal, Québec, Canada; Faculty of Medicine and Health Sciences, McGill University, Montreal, Québec, Canada

## Abstract

Statins are a cornerstone in the medical management of cardiovascular disease, yet their efficacy varies greatly between individuals. In this commentary, we outline the evidence for the role of CD4^+^CD28^null^ T-cell expansion as a critical moderator of the effects of statins in preventing cardiovascular events via the reduction of pathological inflammation. Given this relationship, we argue that T-cell profiles should be considered as a patient characteristic in clinical and preclinical studies examining statin efficacy in other age- and inflammation-related pathologies. We discuss the implications this may have for studies of statin use in numerous disease processes—notably, dementia and neurocognitive dysfunction—and the potential for T-cell profiles to be used as a prognosticator for statin efficacy in rheumatoid arthritis, Alzheimer’s disease, and multiple sclerosis.

## Research Letter

Statins are a commonly used, broad class of medications known to reduce atherosclerosis and prevent severe ischemic cardiovascular disease. Statins reduce cholesterol synthesis by inhibiting 3‐hydroxy‐3‐methyl glutaryl coenzyme A reductase (HMG-CoA reductase), and the clinical benefits of reductions in serum cholesterol are well documented (1). However, recent insights from clinical and preclinical studies have demonstrated that statins have pleiotropic effects and likely also modulate inflammation via numerous mechanisms, such as by inhibiting the activity of inflammatory cytokines and by reducing antigen presentation and the polarization of T cells (2). The efficacy of statins can vary greatly between individuals, with contemporary studies suggesting that their benefits are highly dependent on the initial presence of an expanded CD4^+^CD28^null^ T-cell subpopulation (3). Despite this evidence, the contribution of T-cell profiles to the efficacy of statins is often overlooked in clinical studies, statistically obfuscating the potential benefits of statin therapy present in subsets of patients with aberrant CD4^+^CD28^null^ T-cell expansion. Here, we begin by briefly describing the characteristics of CD4^+^CD28^null^ T cells before reviewing 3 lines of evidence that lead us to conclude that T-cell profile characterization in clinical and preclinical studies may help to elucidate the benefits of statins in numerous age-related diseases—including neurodegenerative diseases.

CD4^+^ T cells play critical roles in regulating immune responses by facilitating inflammation and activating the humoral immune system while coordinating adaptive suppression of the immune reaction. Naïve CD4^+^ T cells are activated by costimulation of T-cell receptor binding with MHC-II on an antigen-presenting cell (APC) and T-cell-bound CD28 with an APC-bound costimulatory molecule. Costimulation also contributes to T-cell regulatory function, being necessary for terminating immune responses and preventing autoimmunity (4). CD4^+^ T cells that lack CD28 are associated with aberrant inflammatory states (5). Although the CD4^+^CD28^null^ subset occupies a very small proportion of the total T-cell population in healthy young adults—estimated to comprise between 0.1% and 2.5% of T cells (6)—this subpopulation is expanded with advanced age, and especially in the context of age-related pathologies (7). Indeed, the CD4^+^CD28^null^ T-cell population can be elevated in age-related disease contexts such as those produced by cardiovascular disease (3), autoimmune disorders such as rheumatoid arthritis (8) and multiple sclerosis (5), as well as in Alzheimer’s disease (AD) (9,10). Thus, an abnormal age-associated loss of costimulatory molecules may have detrimental effects on both the initiation of the immune response as well as its adaptive suppression.

The well-known cardiovascular benefits of statins may have T-cell-dependent effects in addition to their well-understood cardiovascular protective mechanism of decreasing low-density lipoprotein (LDL) (11). Indeed, a seminal investigation showing the efficacy of statin use in reducing recurrent ischemic events in unstable angina patients found that the benefits of statin therapy were highly dependent on the presence of an initial CD4^+^CD28^null^ T-cell expansion (3). When stratifying unstable angina patients by CD4^+^CD28^null^ T-cell expansion status, Liuzzo et al. found that patients with an expansion (>10% of total T cells) saw almost half the number of cardiovascular events after 2 years if they were on statins than if they were not. In contrast, the risk of recurrence of cardiovascular disease remained low for the patients with low (<1%) CD4^+^CD28^null^ T-cell levels regardless of statin use, indicating that statins provided limited cardiovascular benefits to these patients (3). These findings suggest that the CD4^+^CD28^null^ T-cell expansion status is an important predictor of how much a patient may benefit from statin therapy with respect to the prevention of ischemic cardiovascular events.

Further, statins have been shown to induce apoptosis of CD4^+^CD28^null^ T cells. This is a powerful finding considering CD4^+^CD28^null^ T cells show decreased expression of Fas-associated death domain-like IL-1-converting enzyme inhibitory protein (FLIP), thus rendering them resistant to this common apoptotic mechanism in T cells (12). Statins induce apoptosis by reducing the expression of the anti-apoptotic BCL-2 protein in all CD4^+^ T cells, and thus circumvent this resistance to FLIP-mediated apoptosis (13). Presumably, statins help increase pan-CD4^+^ T-cell turnover allowing for the compensatory generation of younger, healthier T-cell populations. A retrospective clinical study found that unstable angina patients treated with statins for at least 1 month had fewer CD4^+^CD28^null^ T cells than those given standard anti-ischemic therapy without statins, an effect that correlated well with plasma C-reactive protein (CRP) levels (14). A separate clinical investigation reported that CD4^+^CD28^null^ T-cell expansion is found in ST-elevation myocardial infarction (STEMI) but not non-ST-elevation myocardial infarction (NSTEMI) patients, and that the counts of these cells were dramatically reduced after 72 hours of rosuvastatin treatment, an effect that persists up to 42 days in patients with initially elevated CD4^+^CD28^null^ T-cell counts (13). Together, these findings suggest that the benefits of statins in the context of ischemic cardiovascular disease may well depend on the initial presence of pro-inflammatory and aberrantly expanded CD4^+^CD28^null^ T-cell population.

The ability of statins to reduce CD4^+^CD28^null^ T-cell levels and dampen pathological inflammatory responses suggests that these drugs may have utility in other inflammatory contexts, including age-related disorders. Indeed, statin use has been a proposed therapeutic avenue for many pathologies with some encouraging results in animal models and human randomized controlled trials (15,16). When assessing statin efficacy, meaningful improvements may be overlooked when pooling all patients together as not all in the sample may have comparable levels of CD4^+^CD28^null^ T-cell expansion. Thus, it may be critical to stratify patients by this parameter in analyses. Indeed, it is possible that statin therapy, as an inflammatory modulator for hyper-reactive T-cell subsets, may have beneficial effects in a myriad of age-related diseases if targeted to patient populations with CD4^+^CD28^null^ expansion. The advantages would be considerable in the context of diseases such as AD, in which neuroimmune dysfunction contributes to disease progression (17), and for which many disease-modifying treatments are nascent, expensive, or unavailable. Due to the abundance of statin drugs and their reasonable safety profile, such a finding could prompt the rapid repurposing of statins for many age-related inflammatory and/or neurodegenerative conditions with relative ease.

A final line of evidence is that aberrantly expanded T cells may explain inconsistent findings in the relationship between statin use and cognitive performance. Studies reporting on the association between statins and cognitive performance are inconsistent—even paradoxical—at 2 levels: between samples of healthy elderly participants and between studies on statin use and dementia. Post-market surveillance research associates statin use in older adults worsened short-term memory and attention (18,19), leading to hypotheses that this association may be causal. However, the relationship between statin use and cognitive performance in AD may be different. Meta-analyses find that statin use may lessen the severity of AD or slow AD-associated neurocognitive decline (20,21), whereas other reviews find little evidence of neurocognitive benefits for AD (22). Mediation of the effects of statins on cognitive function by T-cell population expansions may help explain these inconsistent findings. The prevalence of research participants with CD4^+^CD28^null^ T-cell expansion may well differ between individuals in these various studies. Given the points discussed earlier, statins may have stronger neuroprotective effects, and thus help preserve cognitive function in those with CD4^+^CD28^null^ T-cell expansion. Based on this model, one would hypothesize that statin usage may improve long-term cognitive performance in AD patient subpopulations that have higher baseline inflammatory status but might have short-term negative effects on cognitive performance in patients with more normal T-cell profiles. Indeed, a recent study by Gentreau and colleagues investigated the relationship between statin use and cognitive performance at both acute and chronic time points, finding that statin use was negatively associated with cognitive performance at short time points but not after 8 years of treatment (23). Interestingly, reductions in LDL cholesterol from statin therapy were highly negatively associated with cognitive performance, whereas reductions in CRP, an inflammatory marker, were positively associated with cognitive outcomes. These findings suggest that the potential short-term harms in cognitive performance from statin use may be due to LDL cholesterol-dependent mechanisms, whereas potential long-term benefits of statin use may be related to reduced inflammation, especially in those with elevated levels of inflammation to begin with. Testing such a hypothesis and thus improving our understanding of the potential relationship between the neurocognitive effects of statins and their interaction with CD4^+^CD28^null^ T-cell expansion status requires quantification of T-cell subpopulations and participant stratification based on this quantity.

In summary, the drastic difference in the clinical effects of statin use between patients with high and low CD4^+^CD28^null^ T-cell populations, the relationship that statins and CD4^+^CD28^null^ T-cell populations have with inflammation, and inconsistent findings between studies associating statin use with clinical outcomes (eg, neurocognitive performance) all point to a probable contributing role of CD4^+^CD28^null^ T-cell expansion. We urge that research examining T-cell profiles in the context of diverse inflammatory diseases report on participant statin use so that subgroup analyses can be easily conducted. Given the data earlier, prospective investigations examining the efficacy of statin use on the progression of disease states should, when possible, consider T-cell profiling by flow cytometry to allow for more nuanced drug–immunology interaction analyses to be conducted. Indeed, contemporary flow cytometric analyses can be incorporated into laboratory tests used clinically for more personalized diagnostics and prognostication (24). In addition to improving the field’s understanding of the mechanisms underlying the pleiotropic effects of statins, assessing CD4^+^CD28^null^ expansion status may help personalize statin prescription for those likely to benefit from their use while reducing harm to those who are not likely to benefit from statin use.
